# Supplementary figures and images for: Rotating cell culture system-induced injectable self-assembled microtissues with epidermal stem cells for full-thickness skin repair
Source: PeerJ. 2024 Oct 31;12:e18418. doi: 10.7717/peerj.18418 (PMC11531757; doi:10.7717/peerj.18418)

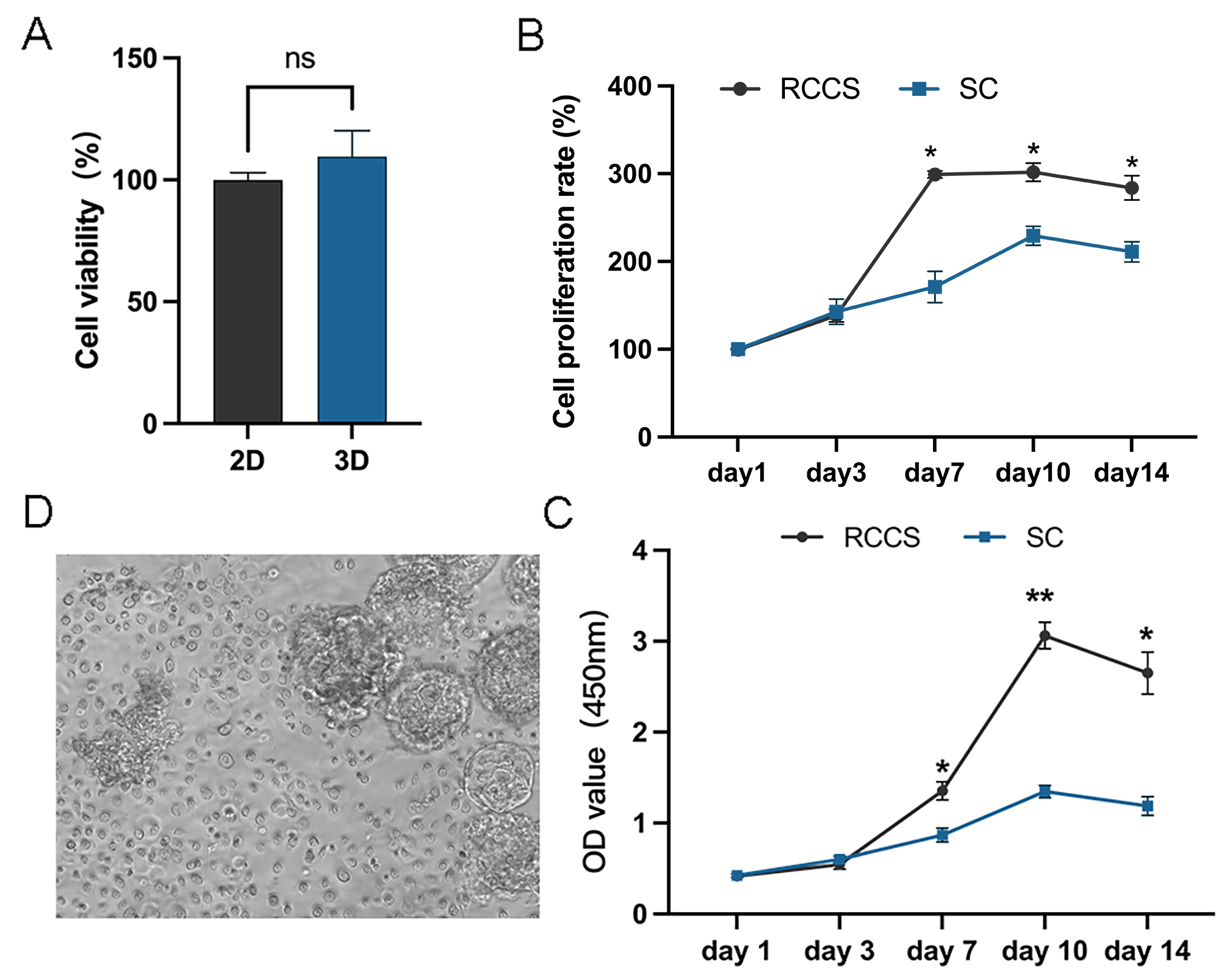

Supplement: Supplemental Information 2 [file peerj-12-18418-s002.jpg]
