# Supplementary material for: Rotating cell culture system-induced injectable self-assembled microtissues with epidermal stem cells for full-thickness skin repair
Source: PeerJ. 2024 Oct 31;12:e18418. doi: 10.7717/peerj.18418 (PMC11531757; doi:10.7717/peerj.18418)
Supplement: Supplemental Information 4 [file peerj-12-18418-s004.pdf]

Table 1. MIQE checklist for authors, reviewers, and editors.		
Item to check	Importance	Section/line number, or reason for not reporting
Experimental design		
Definition of experimental and control groups	E	Line 125
Number within each group	E	Line 126
Assay carried out by the core or investigator's laboratory?	D	
Acknowledgment of authors' contributions	D	
Sample		
Description	E	Line 126
Volume/mass of sample processed	D	
Microdissection or macrodissection	E	Not Applicable
Processing procedure	E	Line 126-127
If frozen, how and how quickly?	E	Line 126-127
If fixed, with what and how quickly?	E	Not Applicable
Sample storage conditions and duration (especially for FFPEb samp	E	Line 126-127
Nucleic acid extraction		
Procedure and/or instrumentation	E	Line 127-128
Name of kit and details of any modifications	E	Line 127-128
Source of additional reagents used	D	
Details of DNase or RNase treatment	E	Line 127-128
Contamination assessment (DNA or RNA)	E	Line 127-128
Nucleic acid quantification	E	Line 127-128
Instrument and method	E	Line 127-128
Purity (A260/A280)	D	
Yield	D	
RNA integrity: method/instrument	E	Line 127-128
RIN/RQI or Cq of 3 and 5 transcripts	E	Not Applicable
Electrophoresis traces	D	
Inhibition testing (Cq dilutions, spike, or other)	E	Not Applicable
Reverse transcription		
Complete reaction conditions	E	Line 128
Amount of RNA and reaction volume	E	Line 128
Priming oligonucleotide (if using GSP) and concentration	E	Not Applicable
Reverse transcriptase and concentration	E	Line 128
Temperature and time	E	Line 128-130
Manufacturer of reagents and catalogue numbers	D	Line 129
Cqs with and without reverse transcription	D	
Storage conditions of Cdna	D	
qPCR target information		
Gene symbol	E	Line 134
Sequence accession number	E	Line 134
Location of amplicon	D	
Amplicon length	E	Not Applicable
In silico specificity screen (BLAST, and so on)	E	Not Applicable
Pseudogenes, retropseudogenes, or other homologs?	D	
Sequence alignment	D	
Secondary structure analysis of amplicon	D	

Location of each primer by exon or intron (if applicable)	E	Not Applicable
What splice variants are targeted?	E	Not Applicable
qPCR oligonucleotides		
Primer sequences	E	Line 134
RTPrimerDB identification number	D	
Probe sequences	D	
Location and identity of any modifications	E	Not Applicable
Manufacturer of oligonucleotides	D	
Purification method	D	
qPCR protocol		
Complete reaction conditions	E	Line 130-132
Reaction volume and amount of cDNA/DNA	E	Line 130
Primer, (probe), Mg2 , and dNTP concentrations	E	Line 130-132
Polymerase identity and concentration	E	Not Applicable
Buffer/kit identity and manufacturer	E	Line 130-132
Exact chemical composition of the buffer	D	
Additives (SYBR Green I, DMSO, and so forth)	E	Line 130-132
Manufacturer of plates/tubes and catalog number	D	
Complete thermocycling parameters	E	Line 130-132
Reaction setup (manual/robotic)	D	
Manufacturer of qPCR instrument	E	Line 130-132
qPCR validation		
Evidence of optimization (from gradients)	D	
Specificity (gel, sequence, melt, or digest)	E	Not Applicable
For SYBR Green I, Cq of the NTC	E	Not Applicable
Calibration curves with slope and y intercept	E	Not Applicable
PCR efficiency calculated from slope	E	Not Applicable
CIs for PCR efficiency or SE	D	
r 2 of calibration curve	E	Not Applicable
Linear dynamic range	E	Not Applicable
Cq variation at LOD	E	Not Applicable
CIs throughout range	D	
Evidence for LOD	E	Not Applicable
If multiplex, efficiency and LOD of each assay	E	Not Applicable
Data analysis		
qPCR analysis program (source, version)	E	Line 132
Method of Cq determination	E	Line 132
Outlier identification and disposition	E	Line 132-133
Results for NTCs	E	Not Applicable
Justification of number and choice of reference genes	E	Line 132
Description of normalization method	E	Line 133
Number and concordance of biological replicates	D	
Number and stage (reverse transcription or qPCR) of technical repli	E	Line 133
Repeatability (intraassay variation)	E	Line 133
Reproducibility (interassay variation, CV)	D	
Power analysis	D	
Statistical methods for results significance	E	Line 180-181
Software (source, version)	E	Line 178

Cq or raw data submission with RDML	D	
